# New Departures in Diagnosis Again

**Published:** 1886-05

**Authors:** 


### Editorial.

### NEW DEPARTURES IN DIAGNOSIS AGAIN.

It is always a matter for serious reflection to determine upon the means of assisting the vision of those whose organs of sight are deficient; and we are particularly embarrassed at present to arrange a telescope for one of our contemporaries, who is unable to see spots upon the sun with his naked eye. That another observer whose visual organs are better formed or better trained has discovered these dark spots is evinced by the following report from the observatory of *Gaillard's Medical Journal* of March:

"Most American readers will be no doubt somewhat surprised to learn that he has had so many of these cases, as he does not seem to have reported them in the medical journals, probably thinking them of but little importance in comparison with the laparotomies for various conditions which he has performed so frequently and with such signal success. As Mr. Tait makes the statement, it must be accepted as correct; but the surprise becomes vastly greater when one reads that he has never met with a single failure, and this notwithstanding the fact that he employs no speculum whatever to aid him in these operations. Such a thing may be possible for Mr. Tait, who is acknowledged by all to possess manual dexterity of no ordinary kind; but it would be interesting to learn whether any other English gynæcologist is accustomed to perform operations for vesico-vaginal fistula in the same manner. Certain it is that it will probably be a long time before our less brilliant American operators will be willing to dispense with the

Sims or other similar speculum in such cases. It is all very well to have eyes on the ends of the fingers, but, if one can have the assistance of the ordinary organ of sight in addition, it is an advantage which is not lightly to be cast aside in operations which are sometimes of the most intricate and delicate character. For the present it must be allowed, to use a perhaps not very classical expression, that the British Lion 'has the bulge' on the Bird of Freedom from a vesico-vaginal point of view."

No one who is familiar with the practice and teaching of Sims, Barnes and Goodell in gynæcology will be imposed upon by the quotations made by the Kansas *Medical Index* in regard to their sanction of any use of the hand that is practicable for diagnostic and operative measures, to the exclusion of instrumental assistance for the ends in view. The latter two stand forth with their living protest against such a distortion of their testimony, and the ghost of Sims, more terrific than that of Banquo, must rise up to condemn such a gross misrepresentation of his appreciation of the speculum and other instrumental aids in detecting and treating disorders of the womb.

The petty device of our contemporary in magnifying our pretentions debars us, even in irony, from allowing any weight to his cavilling at our just criticism upon the erroneous doctrines of two deservedly prominent members of the medical profession, and places the *Index* beyond the pale of enlightened controversy.

